# 5-Amino­pentan-1-ol

**DOI:** 10.1107/S1600536811024731

**Published:** 2011-06-30

**Authors:** Richard Betz, Thomas Gerber, Henk Schalekamp

**Affiliations:** a Nelson Mandela Metropolitan University, Summerstrand Campus, Department of Chemistry, University Way, Summerstrand, PO Box 77000, Port Elizabeth 6031, South Africa

## Abstract

The title compound, C_5_H_13_NO, is an aliphatic amino­alcohol with both functional groups residing on terminal C atoms. Apart from the hy­droxy group, all non-H atoms are nearly co-planar, the maximum deviation of an atom taking part in the least-squares plane defined by the mentioned non-H atoms being 0.029 (1) Å. In the crystal, O—H⋯N and N—H⋯O hydrogen bonds connect the mol­ecules, forming a three-dimensional network.

## Related literature

For the crystal structure of 5-amino­valeric acid, see: Honda *et al.* (1990 ▶). For graph-set analysis of hydrogen bonds, see: Etter *et al.* (1990 ▶); Bernstein *et al.* (1995 ▶).
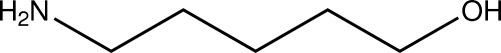


## Experimental

### 

#### Crystal data

C_5_H_13_NO
*M* *_r_* = 103.16
Orthorhombic, 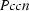

*a* = 10.0973 (2) Å
*b* = 17.4145 (4) Å
*c* = 7.0564 (1) Å
*V* = 1240.79 (4) Å^3^
*Z* = 8
Mo *K*α radiation
μ = 0.08 mm^−1^
*T* = 100 K
0.57 × 0.54 × 0.51 mm

#### Data collection

Bruker APEXII CCD diffractometer
10385 measured reflections
1530 independent reflections
1422 reflections with *I* > 2σ(*I*)
*R*_int_ = 0.030

#### Refinement

*R*[*F* ^2^ > 2σ(*F* ^2^)] = 0.032
*wR*(*F* ^2^) = 0.089
*S* = 1.06
1530 reflections
76 parameters
H atoms treated by a mixture of independent and constrained refinement
Δρ_max_ = 0.38 e Å^−3^
Δρ_min_ = −0.18 e Å^−3^

### 

Data collection: *APEX2* (Bruker, 2010 ▶); cell refinement: *SAINT* (Bruker, 2010 ▶); data reduction: *SAINT*; program(s) used to solve structure: *SIR97* (Altomare *et al.*, 1999 ▶); program(s) used to refine structure: *SHELXL97* (Sheldrick, 2008 ▶); molecular graphics: *ORTEP-3* (Farrugia, 1997 ▶) and *Mercury* (Macrae *et al.*, 2008 ▶); software used to prepare material for publication: *SHELXL97* and *PLATON* (Spek, 2009 ▶).

## Supplementary Material

Crystal structure: contains datablock(s) I, global. DOI: 10.1107/S1600536811024731/ez2250sup1.cif

Supplementary material file. DOI: 10.1107/S1600536811024731/ez2250Isup2.cdx

Structure factors: contains datablock(s) I. DOI: 10.1107/S1600536811024731/ez2250Isup3.hkl

Supplementary material file. DOI: 10.1107/S1600536811024731/ez2250Isup4.cml

Additional supplementary materials:  crystallographic information; 3D view; checkCIF report

## Figures and Tables

**Table 1:** Hydrogen-bond geometry (Å, °)

*D*—H⋯*A*	*D*—H	H⋯*A*	*D*⋯*A*	*D*—H⋯*A*
O1—H81⋯N1^i^	0.879 (14)	1.851 (14)	2.7284 (8)	176.5 (12)
N1—H71⋯O1^ii^	0.886 (13)	2.225 (13)	3.0768 (9)	161.3 (10)
N1—H72⋯O1^iii^	0.881 (12)	2.248 (12)	3.0954 (8)	161.2 (9)

Symmetry codes: (i) 
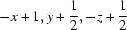
; (ii) 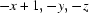; (iii) 
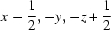
.

**Fig. 1.**
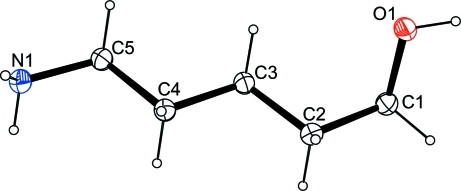
The molecular structure of the title compound, with atom labels and anisotropic displacement ellipsoids (drawn at 50% probability level).

**Fig. 2.**
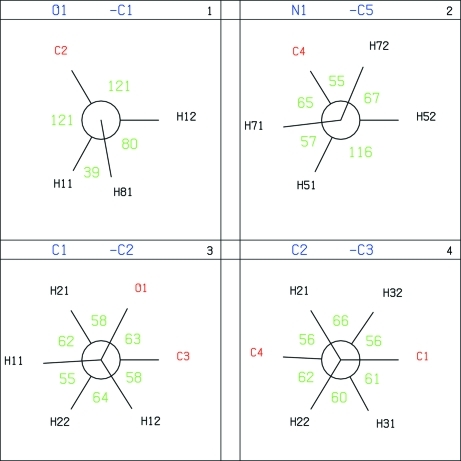
Selected Newman projections and dihedral angles in the title compound.

**Fig. 3.**
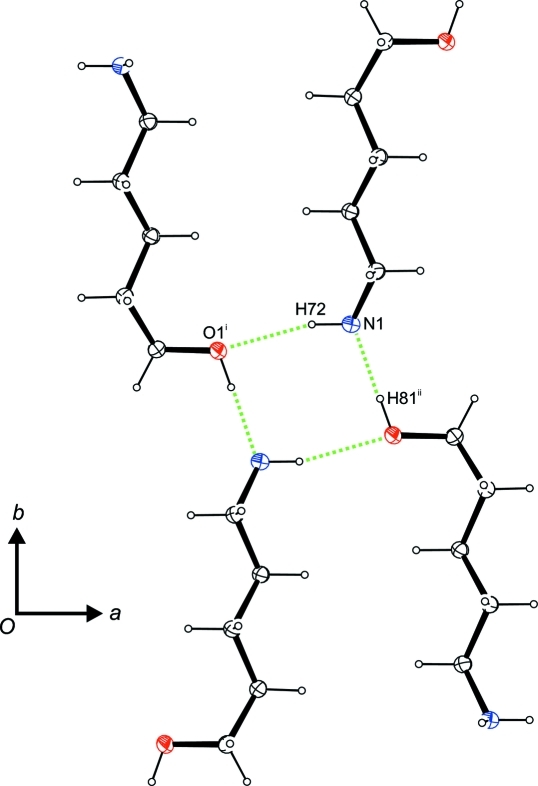
Tetrameric units formed by cooperative hydrogen bonding (indicated by dashed lines), viewed along [0 0 - 1]. Symmetry operators: ix - 1/2, -y, -z + 1/2; ii -x + 1, y - 1/2, -z + 1/2.

**Fig. 4.**
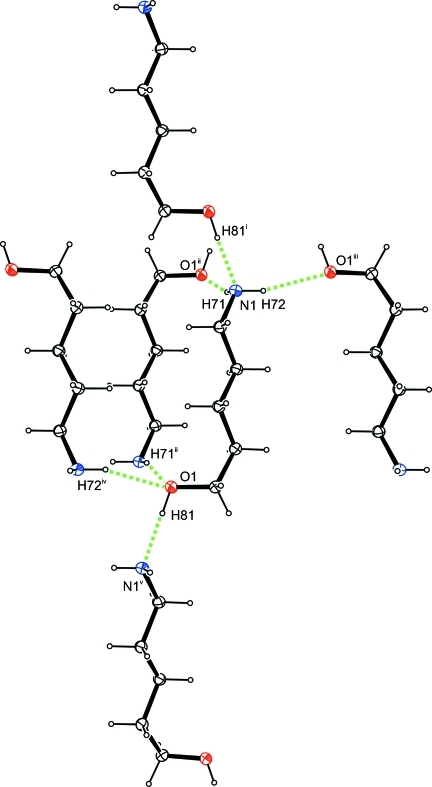
Intermolecular contacts (indicated by dashed lines), viewed approximately along [0 0 - 1]. Symmetry operators: i -x + 1, y - 1/2, -z + 1/2; ii -x + 1, -y, -z; iiix - 1/2, -y, -z + 1/2; ivx + 1/2, -y, -z + 1/2; v -x + 1, y + 1/2, -z + 1/2.

**Fig. 5.**
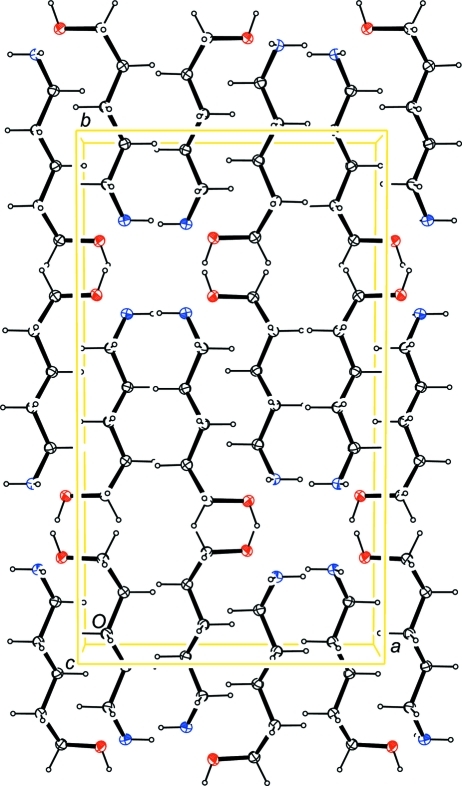
Molecular packing of the title compound, viewed along [0 0 - 1] (anisotropic displacement ellipsoids drawn at 50% probability level).

## supplementary crystallographic information

### Comment

Multidentate ligands are versatile complexation agents for a variety of main group elements as well as transition metals. The possible formation of chelate compounds, in this aspect, is – as a rule of thumb – more likely for smaller chelate ring sizes with four-, five- and six-membered ring systems while the formation of bigger rings is comparatively hampered. As a consequence, the knowledge about the properties of such compounds is limited and precludes a thorough assessment of their properties. In our continuous efforts to elucidate the rules guiding the formation of coordination compounds with chelate ligands featuring a *N*/*O*-set of donor atoms, we determined the crystal structure of the title compound to allow for comparative studies with envisioned coordination compounds. The crystal structure of the oxidation product of the title compound, 5-aminovaleric acid, is apparent in the literature (Honda *et al.*, 1990).

The molecule is a primary alcohol and primary amine at the same time. The molecule adopts a zigzag-chain conformation. Apart from the hydroxy group, all non-hydrogen atoms are in-plane. The OH group encloses a dihedral angle of 62.86 (8) ° with the atoms of the carbon chain (Fig. 1 and Fig. 2).

In the molecule, hydrogen bonds between the hydroxy group as well as the amino groups give rise to a three-dimensional network. While a set of cooperative hydrogen bonds (alternating between hydroxy groups and amino groups) connects the molecules to discrete tetrameric units (Fig. 3), the remaining hydrogen atom of the amino group gives rise to centrosymmetric dimers upon interaction with the hydroxy group. In terms of graph-set analysis (Etter *et al.*, 1990; Bernstein *et al.*, 1995), the hydrogen bonding system requires a *C*^1^_1_(8)*C*^1^_1_(8)*C*^1^_1_(8) descriptor on the unitary level. A description of the cooperative set of hydrogen bonds is possible on the binary level with a *R*^4^_4_(8) descriptor while the centrosymmetric dimers necessitate a *R*^2^_2_(16) descriptor on the unitary level (Fig. 4).

The packing of the compound in the crystal is shown in Figure 5.

### Experimental

The compound was obtained commercially (Aldrich). Crystals suitable for the X-ray diffraction study were taken directly from the provided compound.

### Refinement

Carbon-bound H atoms were placed in calculated positions (C—H 0.99 °) and were included in the refinement in the riding model approximation, with *U*(H) set to 1.2*U*_eq_(C). The hydrogen atoms of the hydroxy as well as the amino group were located on a difference Fourier map and refined freely.

### Crystal data

**Table:**

C_5_H_13_NO	*D*_x_ = 1.105 Mg m^−^^3^
*M**_r_* = 103.16	Melting point = 308–310 K
Orthorhombic, *P**c**c**n*	Mo *K*α radiation, λ = 0.71073 Å
Hall symbol: -P 2ab 2ac	Cell parameters from 8278 reflections
*a* = 10.0973 (2) Å	θ = 2.3–28.2°
*b* = 17.4145 (4) Å	µ = 0.08 mm^−^^1^
*c* = 7.0564 (1) Å	*T* = 100 K
*V* = 1240.79 (4) Å^3^	Block, colourless
*Z* = 8	0.57 × 0.54 × 0.51 mm
*F*(000) = 464	

### Data collection

**Table:**

Bruker APEXII CCD diffractometer	1422 reflections with *I* > 2σ(*I*)
Radiation source: fine-focus sealed tube	*R*_int_ = 0.030
graphite	θ_max_ = 28.3°, θ_min_ = 2.3°
φ and ω scans	*h* = −13→13
10385 measured reflections	*k* = −19→23
1530 independent reflections	*l* = −9→9

### Refinement

**Table:**

Refinement on *F*^2^	Primary atom site location: structure-invariant direct methods
Least-squares matrix: full	Secondary atom site location: difference Fourier map
*R*[*F*^2^ > 2σ(*F*^2^)] = 0.032	Hydrogen site location: inferred from neighbouring sites
*wR*(*F*^2^) = 0.089	H atoms treated by a mixture of independent and constrained refinement
*S* = 1.06	*w* = 1/[σ^2^(*F*_o_^2^) + (0.0468*P*)^2^ + 0.2724*P*] where *P* = (*F*_o_^2^ + 2*F*_c_^2^)/3
1530 reflections	(Δ/σ)_max_ < 0.001
76 parameters	Δρ_max_ = 0.38 e Å^−^^3^
0 restraints	Δρ_min_ = −0.18 e Å^−^^3^

### Fractional atomic coordinates and isotropic or equivalent isotropic displacement parameters (Å^2^)

**Table:**

	*x*	*y*	*z*	*U*_iso_*/*U*_eq_	
O1	0.55733 (5)	0.19608 (3)	0.27542 (8)	0.01969 (16)	
H81	0.5858 (12)	0.2427 (8)	0.3007 (16)	0.040 (3)*	
N1	0.35089 (6)	−0.16146 (3)	0.13148 (9)	0.01779 (16)	
H71	0.3718 (11)	−0.1601 (6)	0.0096 (19)	0.031 (3)*	
H72	0.2639 (12)	−0.1619 (6)	0.1406 (15)	0.027 (3)*	
C1	0.42120 (7)	0.19396 (4)	0.32624 (11)	0.01792 (17)	
H11	0.3781	0.2430	0.2908	0.022*	
H12	0.4124	0.1872	0.4650	0.022*	
C2	0.35429 (7)	0.12773 (4)	0.22406 (10)	0.01683 (17)	
H21	0.3652	0.1347	0.0857	0.020*	
H22	0.2583	0.1288	0.2524	0.020*	
C3	0.41026 (7)	0.04968 (4)	0.28073 (9)	0.01553 (17)	
H31	0.3953	0.0418	0.4180	0.019*	
H32	0.5071	0.0498	0.2587	0.019*	
C4	0.34889 (7)	−0.01724 (4)	0.17219 (10)	0.01532 (17)	
H41	0.2520	−0.0173	0.1934	0.018*	
H42	0.3646	−0.0098	0.0349	0.018*	
C5	0.40539 (7)	−0.09476 (4)	0.23205 (10)	0.01638 (17)	
H51	0.5025	−0.0938	0.2124	0.020*	
H52	0.3894	−0.1016	0.3694	0.020*	

### Atomic displacement parameters (Å^2^)

**Table:**

	*U*^11^	*U*^22^	*U*^33^	*U*^12^	*U*^13^	*U*^23^
O1	0.0152 (3)	0.0157 (3)	0.0281 (3)	−0.00153 (18)	0.00239 (19)	−0.0044 (2)
N1	0.0173 (3)	0.0145 (3)	0.0216 (3)	−0.0007 (2)	0.0004 (2)	−0.0005 (2)
C1	0.0162 (3)	0.0153 (3)	0.0223 (4)	0.0007 (2)	0.0022 (3)	−0.0023 (3)
C2	0.0152 (3)	0.0152 (3)	0.0200 (3)	0.0005 (2)	−0.0014 (2)	0.0004 (2)
C3	0.0157 (3)	0.0143 (3)	0.0165 (3)	−0.0006 (2)	−0.0012 (2)	−0.0003 (2)
C4	0.0153 (3)	0.0142 (3)	0.0165 (3)	−0.0003 (2)	−0.0009 (2)	0.0000 (2)
C5	0.0164 (3)	0.0146 (3)	0.0181 (3)	0.0001 (2)	−0.0013 (2)	0.0007 (2)

### Geometric parameters (Å, °)

**Table:**

O1—C1	1.4210 (8)	C2—H22	0.9900
O1—H81	0.879 (14)	C3—C4	1.5260 (9)
N1—C5	1.4682 (9)	C3—H31	0.9900
N1—H71	0.886 (13)	C3—H32	0.9900
N1—H72	0.881 (12)	C4—C5	1.5252 (9)
C1—C2	1.5188 (9)	C4—H41	0.9900
C1—H11	0.9900	C4—H42	0.9900
C1—H12	0.9900	C5—H51	0.9900
C2—C3	1.5253 (9)	C5—H52	0.9900
C2—H21	0.9900		
			
C1—O1—H81	106.8 (8)	C2—C3—H31	108.9
C5—N1—H71	111.1 (7)	C4—C3—H31	108.9
C5—N1—H72	110.2 (7)	C2—C3—H32	108.9
H71—N1—H72	108.0 (10)	C4—C3—H32	108.9
O1—C1—C2	109.28 (6)	H31—C3—H32	107.7
O1—C1—H11	109.8	C5—C4—C3	112.65 (6)
C2—C1—H11	109.8	C5—C4—H41	109.1
O1—C1—H12	109.8	C3—C4—H41	109.1
C2—C1—H12	109.8	C5—C4—H42	109.1
H11—C1—H12	108.3	C3—C4—H42	109.1
C1—C2—C3	112.80 (6)	H41—C4—H42	107.8
C1—C2—H21	109.0	N1—C5—C4	115.23 (6)
C3—C2—H21	109.0	N1—C5—H51	108.5
C1—C2—H22	109.0	C4—C5—H51	108.5
C3—C2—H22	109.0	N1—C5—H52	108.5
H21—C2—H22	107.8	C4—C5—H52	108.5
C2—C3—C4	113.48 (6)	H51—C5—H52	107.5
			
O1—C1—C2—C3	62.86 (8)	C2—C3—C4—C5	−179.54 (6)
C1—C2—C3—C4	−177.13 (6)	C3—C4—C5—N1	−179.56 (6)

### Hydrogen-bond geometry (Å, °)

**Table:**

*D*—H···*A*	*D*—H	H···*A*	*D*···*A*	*D*—H···*A*
O1—H81···N1^i^	0.879 (14)	1.851 (14)	2.7284 (8)	176.5 (12)
N1—H71···O1^ii^	0.886 (13)	2.225 (13)	3.0768 (9)	161.3 (10)
N1—H72···O1^iii^	0.881 (12)	2.248 (12)	3.0954 (8)	161.2 (9)

Symmetry codes: (i) −*x*+1, *y*+1/2, −*z*+1/2; (ii) −*x*+1, −*y*, −*z*; (iii) *x*−1/2, −*y*, −*z*+1/2.
